# Sternal Abnormalities on Thoracic Radiographs of Dogs and Cats

**DOI:** 10.3390/ani13071233

**Published:** 2023-04-02

**Authors:** Dirk H. N. van den Broek, Siemone C. Vester, Mauricio Tobón Restrepo, Stefanie Veraa

**Affiliations:** Department of Clinical Sciences, Faculty of Veterinary Medicine, Utrecht University, Yalelaan 108, 3584 CM Utrecht, The Netherlands

**Keywords:** pectus excavatum, pectus carinatum, dislocation, vacuum phenomenon, osteoarthrosis, malformation, companion animals

## Abstract

**Simple Summary:**

The chest bone, or sternum, protects the heart and lungs and aids in breathing motion. It is included on chest radiographs of dogs and cats, but little information is available for veterinarians on what abnormalities or diseases affect the chest bone in companion animals. We reassessed chest radiographs of dogs and cats taken in our hospital over a 2 year period to describe these changes. We found that abnormalities of the chest bone were visible in 24% of dogs and 29% of cats, with the most common abnormality being age-related degeneration. Most of the abnormalities noted were of minor clinical importance, but in some animals, conditions that could be painful or otherwise affect well-being were seen.

**Abstract:**

Evaluation of the sternum is part of the routine examination of small animal thoracic radiographs. However, descriptions on frequency and type of abnormalities are lacking. This retrospective observational study aimed to describe abnormal radiographic findings of the sternum in a cross-section of client-owned dogs and cats undergoing thoracic radiography between 1 January 2019 and 1 January 2021 for reasons unrelated to diseases of the sternum. The study population consisted of 777 dogs (mean age, 7.3 ± 3.9 years) and 183 cats (mean age, 7.3 ± 5.1 years). Sternal abnormalities were observed in 189/777 (24%) dogs and 53/183 (29%) cats, mostly around the intersternebral cartilages, accounting for 32/80 (40%) dogs and 20/35 (57%) cats. This was followed by an abnormal number of sternal segments (8% dogs, range 3–9 sternebrae; 15% cats, range 7–9 sternebra). Pectus excavatum was observed in 6/777 (0.8%) dogs and 6/183 (3%) cats, and pectus carinatum in 18/777 (2%) dogs and 2/183 (1%) cats. Post-traumatic changes, such as subluxation, were observed in nine dogs (1.1%) and three cats (1.6%). Presumed prostatic carcinoma metastasis and malignant lymphoma were observed in two dogs (0.2%). Incidental radiographic sternal abnormalities are common in cats and dogs but mostly of unknown clinical relevance.

## 1. Introduction

An evaluation of the sternum is part of the routine examination of small animal thoracic radiographs, as it forms the ventral contour of the thoracic cavity. The sternum contributes to the bony protection of the intra-thoracic cardiopulmonary structures, as well as to the stability and breathing motion of the thorax.

The small animal sternum has been defined as an unpaired segmented series of typically eight bones, called sternebrae [1,2,3,4]. The cranial first sternebra, or manubrium of the sternum (*manubrium sterni*), is wider and longer than the other sternal segments. The manubrium of the sternum is the point of insertion of the sternocephalicus muscle and has lateral shelves of bone accommodating the attachment of the costal cartilages of the first pair of ribs. The body of the sternum (*corpus sterni*) consists of six rectangular to cylindrical-shaped sternebrae. The most caudal last sternebra, or xiphoid process (*processus xiphoideus*), is flat and long, occasionally with a foramen in its caudal half. The xiphoid process is prolonged caudally by the xiphoid cartilage (*cartilago xiphoidea*), which supports the cranial part of the ventral abdominal wall and from which the linea alba extends caudally to the symphysis pelvis. The individual sternebrae are connected by intersternebral cartilages, forming cartilaginous joints (*synchondroses sternales*), and the sternum is covered on the ventral and dorsal surface by thickened periosteum, forming the sternal membrane (*membrana sterni*). Whilst the costal cartilages of the first pair of ribs articulate directly with the manubrium of the sternum, the costal cartilages of the second to seventh ribs articulate with the consecutive intersternebral cartilages between the individual sternal segments, and both the costal cartilages of the eighth and ninth rib pairs articulate with the intersternebral cartilage between the seventh sternebra and the xiphoid process. The first eight paired sternocostal joints are synovial joints, but no synovial joint was found at the ninth sternocostal articulation The costal cartilages of the last four pairs of ribs do not directly articulate with the sternum but connect via the costal arch or are floating ribs, and these ribs are therefore considered asternal ribs [1,2,3,4].

Species differences in the shape of the sternum exist. In dogs the sternum is curved and the sternebrae of the body of the sternum are rectangular in form, with the height exceeding the width. In cats, the shape of the sternum is described as a straight and uniform cylinder [4,5]. On thoracic radiographs, only the sternebrae are visible as individual structures because their high mineral content results in increased x-ray beam attenuation, and therefore higher opacity, on the digital image. The soft tissue structures of the sternum, such as the intersternebral cartilages, are generally not individually identifiable on a radiograph because of border effacement with the neighboring soft tissue of the thoracic wall [6].

Congenital malformation of the sternum can consist of dorsal or ventral deviations (pectus excavatum and pectus carinatum, respectively), as well as numeric or shape changes, such as cleft sternum. Sternal malformations sometimes form a part of a larger congenital defect with expansion to the adjacent thoracic and abdominal structures as well as diaphragmatic cupula [7,8,9,10,11,12,13]. Acquired sternal abnormalities include neoplasia [14], traumatic or pathological fracture and luxation [15,16,17], infection [18,19,20], and degenerative disease of the intersternebral cartilage [21].

No reports on occurrence of sternal abnormalities, either congenital or acquired, as a part of routine radiographic thoracic evaluation are available in dogs and cats. Although not all congenital variations in sternal conformation might be clinically relevant, some of these malformations and abnormalities are causing clinical signs. Being familiar with these abnormalities and the normal variation present, is therefore considered essential in image interpretation. The aim of this study was to describe the occurrence of sternal abnormalities on routine thoracic radiographs of dogs and cats.

## 2. Materials and Methods

This observational study had a cross-sectional and retrospective design. Thoracic radiographic examinations of dogs and cats performed between 1 January 2019 and 1 January 2021 were retrieved from the Picture Archiving and Communication System (Agfa Healthcare Enterprise Imaging, Agfa Healthcare N.V., Belgium) of the Division of Diagnostic Imaging of the Faculty of Veterinary Medicine, Utrecht University, The Netherlands. Images from client-owned animals that had thoracic radiography performed for reasons unrelated to diseases of the sternum were used with anonymity preserved. Examinations were excluded if the thoracic radiographs did not include the entire sternum in the field of view. For animals with repeat examinations performed within the study period, only the first performed radiographic study of the thorax was included for analysis.

The thoracic radiographic studies were obtained with a direct radiography system (Optimus NZR136, Philips, Best, The Netherlands) using a radiographic technique optimized for examination of the thorax (i.e., exposure during maximum inspiration and high kV, low mAs technique) with exposure factors based on patient body size, typically 105–110 kV; 1.2–1.4 mAs for dogs and 50 kV; 5 mAs for cats. A grid was used if object thickness was >10 cm. Animals were not routinely sedated and were positioned using manual restraint by trained personnel wearing protective clothing and personal dosimeters.

Assessment of the sternum was performed on left lateral and dorsoventral thoracic radiographs displayed on medical-grade greyscale monitors (Coronis Fusion 6MP DL, Barco, Kortrijk, Belgium). Images were independently reviewed by two European College of Veterinary Diagnostic Imaging resident (DHNB and SCV) and two European College of Veterinary Diagnostic Imaging diplomates (MTR and SV). For each animal, the sternum was subjectively assessed on alignment, number, shape, and opacity of sternebrae, and width and opacity of the intersternebral cartilages on orthogonal radiographs. A total number of eight sternebrae, including the manubrium of the sternum and xiphoid process, was considered the normal number of sternal segments in both cats and dogs [1,3], and any animal with a higher or lower number of sternal segments was considered abnormal for the purpose of this study.

The vertebral index was calculated as a measure of severity of pectus excavatum using the vertebra overlying the deviation at the most depressed point of the thoracic wall [11,12,22]. The severity of pectus excavatum was characterized as mild if the vertebral index was >9, moderate if the vertebral index was 6–8.99, and severe if the vertebral index was <6, as previously described [11,23]. The type of pectus excavatum was determined based on the anatomical location of the deformity of the sternum and rib cartilage. Pectus excavatum was characterized as the typical form when the condition affected the caudal sternum from the 5th to 8th sternebrae and as an atypical form when the dorsal deviation was noted in the cranial to mid-region of the sternum [10,22].

Numerical data are reported as mean (standard deviation [SD]) if normally distributed or as median (range) if the distribution was skewed. Data were analyzed using IBM SPSS Statistics (Version 28). Independent samples *t*-test was used to assess differences in age between groups of animals with and without degenerative changes affecting the sternum. Statistical significance was set at *p* < 0.05.

## 3. Results

### 3.1. Dogs

**Figure 1:**
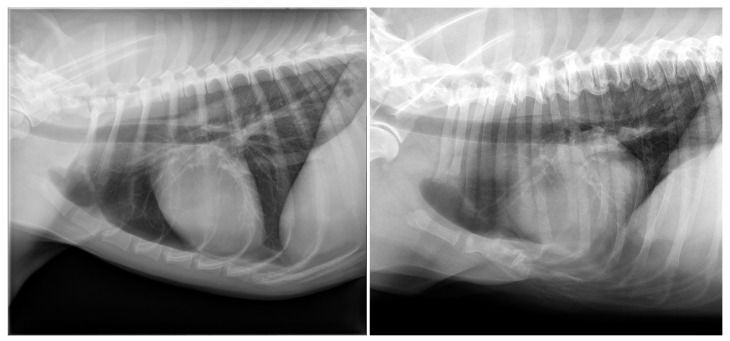
(**Left**) panel: Example of a left lateral radiograph of a dog with a sternum that was considered normal. (**Right**) panel: Abnormally short sternum in a 9-year-old, female neutered Bearded Collie. Caudad to the manubrium of the sternum, fusion of two to three shortened sternebrae with complete absence of the caudal part of the sternum is noted. The diaphragm shows cranial excursion, and the cardiac silhouette is mildly dorsally displaced. Vacuum phenomenon is visible between the manubrium of the sternum and second sternebra.

The study population consisted of 777 dogs. The group of dogs had a mean age of 7.3 (SD, 3.9) years and consisted of 400 males (188 neutered) and 377 females (252 neutered) of various breeds, the most common being mixed breed (*n* = 145), Labrador Retriever (*n* = 62), Chihuahua (*n* = 28), Bernese Mountain Dog (*n* = 26), Golden Retriever (*n* = 23), French Bulldog (*n* = 23), Labradoodle (*n* = 22), and German Shepherd (*n* = 21). One hundred and fourteen dogs were of brachycephalic breed. The three most common indications for thoracic radiography in dogs were metastasis screening (*n* = 252), cough (*n* = 94), and dysphagia (*n* = 57). An example of a thoracic radiographs of a dog on which the sternum was considered unremarkable is provided in Figure 1.

Sternal abnormalities were observed in 189/777 (24%) dogs (Table 1). Nineteen dogs had more than one sternal abnormality, being degenerative changes in combination with abnormal number of sternebrae (*n* = 8), post-traumatic changes (*n* = 5) or shape deformity of the sternum (*n* = 1), and an abnormal number of sternebrae in combination with shape deformity (*n* = 4) or post-traumatic changes (*n* = 1).

**Figure 2:**
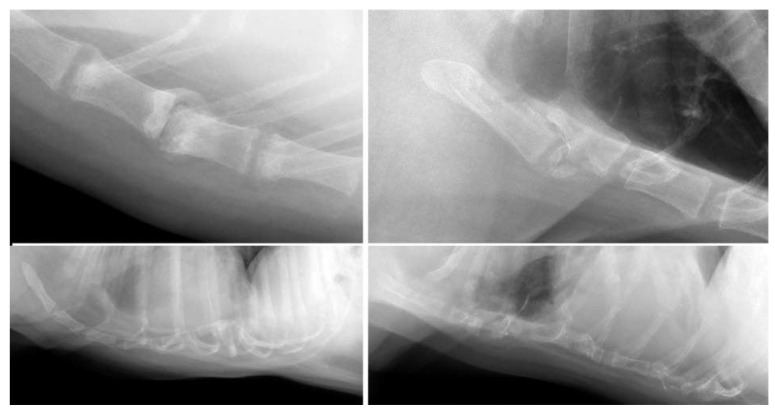
(**Top left**) panel: Degeneration of the intersternebral joint space in an 11-year-old, neutered female Welsh Springer Spaniel. The intersternebral cartilage space between the 4th and 5th sternebrae is narrowed, shows vacuum phenomenon, and is surrounded by new bone formation. The 5th sternebra is mildly displaced ventrally relative to the fourth sternebra. (**Bottom left**): Pectus excavatum in a 12-year-old male Pug. Dorsal deviation was noted in the caudal part of the sternum with border effacement of the ventral thoracic diaphragmatic surface and the caudal ventral cardiac silhouette and an altered position of the heart. (**Top right**): Luxation of the second sternebra in a 10-year-old male neutered mixed-breed dog. The second sternebra is displaced in craniodorsal direction and is overriding the manubrium of the sternum. (**Bottom right**): Pathologic fractures of multiple sternebrae in 10-year-old male Chihuahua with malignant lymphoma.

Abnormalities were most frequently observed in the area of the intersternebral cartilages in dogs (*n* = 108; 14%), most of which were considered degenerative type changes such as mineralisation of the sternal cartilages or formation of bony spurs (*n* = 98; 13%). Marked degenerative changes, such as narrowing of the intersternebral cartilage space (*n* = 13), vacuum phenomenon (*n* = 13), and subluxation (*n* = 5), were mainly observed in the mid-section of the sternum, between the 3rd and 4th and 4th and 5th sternebrae (Figure 1 and Figure 2). Predominantly medium to large breed dogs were affected, with Labrador Retriever as the most common breed (*n* = 16). The mean age of dogs with degenerative changes (mean, 9.8; SD, 2.5 years) was significantly higher than the mean age of dogs without these changes (mean, 6.9; SD, 3.9 years; *p* <0.001).

An abnormal number of sternal segments was the second most seen abnormality in dogs (*n* = 62; 8%). Less than eight sternal segments were observed 46 dogs (6%): seven sternebrae were visible in 40 dogs, six sternebrae in four dogs, and three and four sternebrae in one dog each. The latter resulted in morphologically abnormal short sternum (Figure 2). The lower number of sternebrae predominantly affected small breed dogs, including four out of seven Pugs (57%), five out of fourteen Pomeranians (36%), and six of twenty-eight Chihuahuas (21%) in the study population. In eight dogs, the lower number was due to fusion of two (*n* = 5) or four (*n* = 2) sternebrae. Three dogs with lower number of sternebrae showed border effacement of the ventral thoracic diaphragmatic surface and the caudal ventral cardiac silhouette due to cranial excursion of the diaphragm, but none of these animals had evidence of peritoneopericardial herniation. Supernumerary sternal segments (9 sternebrae in all) were observed in 16 dogs (2%), affecting predominantly medium and large breed dogs. The most common breed affected was Labrador Retriever (*n* = 7).

Pectus excavatum was observed in six dogs (0.8%), and the severity was considered mild in five dogs and moderate in one dog based on vertebral indices (median vertebral index, 10.5; range, 8.6–11.2). Pectus excavatum involved the caudal sternum in five cases and the mid-sternum in one mixed breed dog. One case was considered likely to have a traumatic instead of congenital origin based on clinical history. Four of the five dogs with suspect congenital pectus excavatum were brachycephalic breeds, including two out of seven Pugs (Figure 2). Mild pectus carinatum was observed in 18 dogs (2%) and affected the caudal part of the sternum in all cases. French Bulldog (*n* = 8) and Chihuahua (*n* = 5) were overrepresented.

Presumed post-traumatic changes were observed in 12 dogs (1.5%), although none of these dogs were presented for thoracic radiography because of a history of trauma or suspect traumatic lesions of the sternum; the indication in eight of these dogs was metastasis screening. The presumed post-traumatic changes mainly consisted of dislocation (*n* = 9, of which five were in combination with other degenerative changes) (Figure 2). These changes affected predominantly the mid-section of the sternum. Changes compatible with chronic fracture of sternebrae were observed in two dogs.

One Chihuahua with malignant lymphoma had multiple pathological fractures associated with osteopenia (Figure 2). An aggressive bone lesion, suspect prostatic carcinoma metastasis affecting the second and third sternebrae, was observed in another dog.

### 3.2. Cats

**Figure 3:**
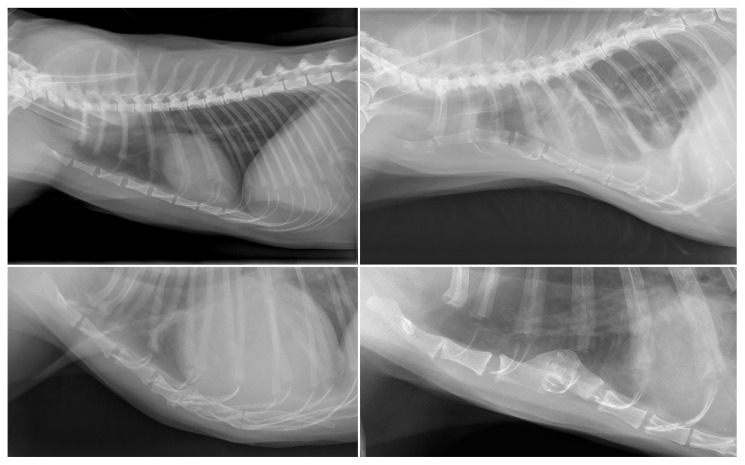
(**Top left**) panel: Example of a left lateral radiograph of a cat with a sternum that was considered normal. (**Bottom left**): Abnormal low number of sternebrae in a 12-year-old female Domestic Shorthair. A total of six individual sternebrae are present, caused by fusion of the fifth and sixth sternebrae and an absence of the seventh sternebra. (**Top right**): Pectus excavatum in an 11-year-old female neutered Domestic Shorthair. (**Bottom right**): Chronic subluxation of the fourth sternebra in an 11-year-old male neutered Domestic Shorthair. The fourth sternebra is displaced dorsally and fused with the third sternebra.

The study population consisted of 183 cats. The group of cats had a mean age of 7.3 (SD, 5.1) years and consisted of 105 males (89 neutered) and 78 females (66 neutered) of various breeds, most commonly Domestic Shorthair (*n* = 112), Maine Coon (*n* = 16), mixed breed (*n* = 11), and British Shorthair (*n* = 10). The most common indications for thoracic radiography in cats were dyspnea (*n* = 40), metastasis screening (*n* = 37), and post-accident trauma (*n* = 18). An example of a thoracic radiographs of a cat on which the sternum was considered unremarkable is provided in Figure 3. 

Sternal abnormalities were observed in 53/183 (29%) cats (Table 2). Six cats had more than one sternal abnormality, being abnormal number of sternebrae in combination with pectus excavatum (*n* = 3) or degenerative changes (*n* = 3).

Degenerative changes, consistent of mineralization of the sternal cartilages or formation of bony spurs, were observed in 15 cats (8%). Cats with degenerative changes were significantly older than those without (mean, 11.2; SD, 5.6 years versus mean, 6.9; SD, 4.9 years; *p* = 0.002). Concurrent gas opacity in the intersternebral cartilage space or associated subluxation of sternebrae was not observed in any of the cats with signs of degenerative changes of the intersternebral cartilages.

An abnormal number of sternebrae was observed in 27 cats (15%), of which seven cats had nine sternebrae. Nineteen cats had seven individual sternal segments, which, in ten cats was caused by complete fusion of the first and second sternebrae (*n* = 5) or sixth and seventh sternebrae (*n* = 5). The other nine cats with seven sternebrae had an absence of one sternal segment. One Domestic Shorthair had six individual sternebrae, caused by complete fusion of the fourth and fifth sternebrae and the absence of one sternal segment (Figure 3).

Pectus excavatum was observed in six cats (3%), and the severity was considered mild in four cats and moderate and severe in one cat each (median vertebral index, 9.8; range, 5.8–11.4) (Figure 3). The typical form of pectus excavatum, affecting the caudal sternum, was observed in 2/6 cats and the atypical form, affecting the cranial or mid-region of the sternum, was observed in 4/6 cats. Mild forms of pectus carinatum were observed in two cats (1%).

Signs of traumatic luxation were observed in three cats (1%). One cat had undergone recent cardiopulmonary resuscitation, and the other two cases showed more chronic changes with malformed fusion of the dislocated sternebrae. One of these cats had a history of sternal fracture 10 years prior and the other cat had no known history of trauma (Figure 3). Other abnormalities observed in cats were a kinked manubrium of the sternum (*n* = 2), partial fusion of sternebrae (*n* = 3), and narrowing of the intersternebral cartilage space without signs of degeneration (*n* = 1).

## 4. Discussion

The sternum develops from the fusion of bilateral mesodermal bars which unite from day 25 of gestation in dogs and day 28 of gestation in cats, starting cranially at the manubrium of the sternum and ending caudally at the xiphoid process [1,24]. Ossification starts at day 40 in dogs but is inhibited at rib attachment sites, resulting in the individual sternebrae with interposed intersternebral cartilages in those areas where the ribs attach. However, this is not the case for the first rib pair, which connects to the ossified manubrium of the sternum but attaches at a later time than the other costal cartilages during development. Nonunion of the caudal part of the sternal bars, anomalous ribs, and uneven apposition of ribs can lead to various morphological abnormalities of the body of the sternum and its individual sternebrae [1]. Sternal defects have been reported with concurrent congenital midline anomalies, such as peritoneopericardial diaphragmatic hernia and cranioventral abdominal wall hernia in dogs and cats [7,8,9,13,25,26]. Abnormal number, fusion, and shape alterations of sternebrae were observed in both cats and dogs in our study population, but in none of these animals was clear evidence of bifid sternum or other ventral abdominal wall anomalies seen. This could in part be explained by the limited visibility of the sternum on straight dorsoventral radiographic projections, caused by superimposition of the vertebral column on the sternum. In addition, mineralized rib cartilage was often superimposed on the most caudal aspect of the sternum on left lateral views. Oblique dorsoventral radiographic views and computed tomography would be more sensitive techniques to identify cleft sternum and concurrent ventral abdominal wall defects [5].

As in previous reports, pectus excavatum mostly affected the caudal sternum in dogs in our study population, which is the typical form of pectus excavatum [12]. Komsta and colleagues (2019) observed pectus excavatum in a large percentage of brachycephalic dogs (44.4%) [10], whilst we observed this condition in only 3.5% of brachycephalic dogs and 0.8% of dogs of all breeds. In that study, Maltese and English Bulldog breed dogs were overrepresented, but in our study population none of the three dogs for each of these two breeds showed evidence of pectus excavatum. A large proportion (67%) of affected cats in our study population had an atypical location of pectus excavatum, in which the dorsal deviation affects the cranial and mid-section of the sternum. This was also reported in a recent publication on computed tomographic features of pectus excavatum in cats, in which half the cats had the atypical form of the disorder [11]. That study found mild pectus excavatum in only 21.5% of cats, whilst most of the cats included had moderate or severe pectus excavatum. In contrast, a larger proportion of pectus excavatum was considered mild in cats in the present study (67%). In part, this could be explained by the fact that none of the cats included in our study were presented for radiographic examination because of complaints related to their thoracic malformation. However, although a strong positive correlation has been reported in brachycephalic dogs with the atypical form of pectus excavatum, no clear association has been found between the radiographic classification and the severity of clinical signs in brachycephalic dogs with the typical form of pectus excavatum [12] or in a group of kittens with pectus excavatum [27]. Moreover, it should be noted that our results are based on thoracic radiographs made on a single time point for each patient, whilst motion of the sternum in severely dyspneic animals could mimic conditions such as pectus excavatum [28]. Therefore, the number of animals with abnormal alignment of the sternum could be overestimated in our study population.

In agreement with a previous publication by Hassan and colleagues (2018), we observed pectus carinatum predominantly in small breed dogs, except for in one English Springer Spaniel [12]. In that previous report, the Pug and French Bulldog were at increased risk of this disorder, with 41% and 18% of the dogs of these breeds affected, respectively. In our study population French Bulldog and Chihuahua were the most common breeds with pectus carinatum, with 8/23 (35%) and 5/28 (18%) animals affected, respectively. Only 1/7 (14%) Pugs showed radiographic evidence of pectus carinatum in our study.

Although neoplastic disease was the most common indication for thoracic radiography in dogs, only two cases presented with radiographic aggressive bone lesions affecting the sternum. One dog diagnosed with malignant lymphoma presented with pathological fractures of multiple sternebrae, the other dog was diagnosed with prostatic carcinoma and had an aggressive bone lesion affecting the second and third sternebrae. However, the sternal lesions were not biopsied in these dogs and therefore it remains unknown if these aggressive bone lesions indeed were caused by neoplastic disease. Skeletal involvement affecting the spine has been described in canine multicentric lymphoma [29,30], but to the author’s knowledge it was not specifically affecting the sternum. Skeletal metastases are common in canine prostatic carcinoma, often accompanied by extraskeletal metastases, and the lumbar vertebra, pelvis, and proximal femur are mostly affected [31]. Metastases to the sternum from prostatic carcinoma appear uncommon, but have been documented in a dog [32]. In a necropsy study, metastatic lesions were observed in the sternum in nine out of twenty-one dogs with metastatic or multicentric tumors with bone involvement, and mammary carcinoma and lung carcinoma were the most common primary tumors [14]. Only one dog with prostatic carcinoma was included in that study, which did not have evidence of bone metastasis. Sternal osteomyelitis, caused by foreign body, systemic mycosis, following sternotomy or hematogenous spread of bacteria, is the main differential diagnosis to neoplasia for sternal aggressive bone lesions [18,19,20,33,34]. The dogs with aggressive bone lesions affecting the sternum included in this study had no other clinical signs or history indicative of infectious disease, but sternal osteomyelitis cannot be completely ruled out because the specific lesions were not biopsied.

Age-related degeneration of the *synchondroses sternales*, mostly consistent of mineralization of the sternal cartilages or formation of bony spurs, was the most common finding in our study, with a prevalence of 14% in dogs and 8% in cats. In 13 of the dogs affected, gas opacity was observed in between the sternebrae on the left lateral view radiograph. Gas accumulation within synovial joints and intervertebral disc spaces is termed vacuum phenomenon. This gas consists of 90–92% nitrogen and its presence has been associated with intervertebral disc degeneration in dogs [35,36,37]. The sternal vacuum phenomenon has previously been described in three dogs [21] and, in agreement with our findings, this was reported in large breed dogs, frequently surrounding the fourth sternebra, and was associated with intersternebral joint space narrowing. Those authors proposed laxity of the intersternebral fibrocartilage and tractile forces caused by radiographic positioning as potential causes for vacuum phenomenon in the intersternebral cartilage space or superimposed sternocostal joints [21]. In our population, vacuum phenomenon was only observed in the intersternebral cartilage space in conjunction with other degenerative changes, such as narrowing of the intersternebral cartilage space, new bone formation or even dislocation of sternebrae. Gas opacity in the intersternebral cartilage space could therefore be considered a sign of degeneration of the *synchondrosis sternalis* in dogs, similar to the above-mentioned association between vacuum phenomenon in the intervertebral disc space and intervertebral disc degeneration in dogs [36,37].

Sternal dislocation is infrequently reported in veterinary literature and is most often considered to be of traumatic origin [15,16,17]. Based on history and other findings, a traumatic origin was suspected in all three cats with sternal dislocation included in our study population. In five dogs, marked degenerative changes of the intersternebral joints coincided with sternal subluxation, which might suggest spontaneous dislocation following instability or arthropathy of these joints. However, due to the cross-sectional study design, it cannot be determined if dislocation preceded or followed intersternebral joint degeneration signs in these dogs. Future research is warranted to examine if sternal degeneration, now considered an incidental finding, affects animal well-being.

Our study had several limitations. First, the retrospective design prohibits adequate correlation between radiographic findings and clinical signs. For example, it cannot be reliably determined if sternal dislocation in dogs with severe intersternebral cartilage degeneration was associated with pain or other clinical signs, because no specific history and physical examination addressing these findings were performed in these cases. Secondly, the large variety in breeds in combination with the relatively low number of cases for each disorder impedes drawing firm conclusions on any breed predispositions. Thirdly, the use of radiography instead of a cross-sectional technique such as computed tomography results in a lower sensitivity for detection of abnormalities because of the inferior contrast resolution and problems of superimposition inherent to radiography. Moreover, computed tomography would allow a better representation of the anatomy. For instance, computed tomography was reported to be useful for evaluation of thoracic asymmetry and sternal torsion in cats with pectus excavatum, which cannot be done with radiography [11]. In addition, the use of intravenous iodinated contrast medium in computed tomography would allow for better characterization of potential soft tissue abnormalities, such as inflammation [18].

## 5. Conclusions

Sternal abnormalities are common incidental findings on thoracic radiographs of companion animals and were observed on thoracic radiographs of 24% of dogs and of 29% of cats in our study population. Clinically relevant abnormalities, such as severe degeneration of the intersternebral cartilage space, dislocation, or aggressive bone lesions, were only recognized in few cases, whilst most animals showed abnormalities that were unlikely to cause clinical complaints.

## Figures and Tables

**Table 1:** Radiographic findings and breeds per category of sternal abnormality in dogs.

Group	Findings	Breeds *
Degeneration (*n* = 98)	New bone formation (*n* = 98); collapse (*n* = 13); vacuum phenomenon (*n* = 13); sclerosis (*n* = 9); dislocation (*n* = 5)	Labrador Retriever (16/62); mixed (13/145); Boxer (7/13); Flatcoated Retriever (7/15); Cavalier King Charles Spaniel (5/11); German Shepherd (5/21); Bernese Mountain Dog (4/26); Belgian Shepherd (3/8); Dachshund (3/19); English Cocker Spaniel (3/13); American Bulldog (2/6); Drentsche Patrijshond (2/6); Golden Retriever (2/23); Irish Setter (2/3); Labradoodle (2/22); Welsh Springer Spaniel (2/3); White Swiss Shepherd Dog (2/14); Anatolian Shepherd Dog (1/1); Border Collie (1/10); Border Terrier (1/4); Bouvier des Flandres (1/2); Chow Chow (1/3); English Springer Spaniel (1/4); Frisian Water Dog (1/2); Hovawart (1/2); Irish Wolfhound (1/2); Jack Russell Terrier (1/15); Kooikerhondje (1/5); Newfoundland (1/5); Dobermann (1/2); Rottweiler (1/10); Spanish Water Dog (1/3); Stabyhoun (1/12); West Highland White Terrier (1/3); Wirehaired Pointing Griffon (1/1)
Abnormal number (*n* = 62)	9 sternebrae (*n* = 16)	Labrador Retriever (7/62); Dachshund (2/19); Beagle (1/7); Australian shepherd (1/4); English Cocker Spaniel (1/13); Irish Wolfhound (1/2); mixed (1/145); Rhodesian Ridgeback (1/9); Whippet (1/5)
	<8 sternebrae (*n* = 46)	Mixed (10/145); Chihuahua (6/28); Pomeranian (5/14); Labrador Retriever (4/62); Pug (4/7); French Bulldog (3/23); Staffordshire Bull Terrier (2/10); West Highland White Terrier (2/3); American Staffordshire Terrier (1/8); Australian Shepherd (1/4); Bearded Collie (1/2); Boxer (1/13); Drentsche Patrijshond (1/6); Great Dane (1/2); Miniature Pinscher (1/1); Dobermann (1/2); Shih Tzu (1/6); Spanish Water dog (1/3)
Shape deformity (*n* = 24)	Pectus excavatum (*n* = 6)	Pug (2/7); Boxer (1/13); Dachshund (1/19); French Bulldog (1/23); mixed (1/145)
	Pectus carinatum (*n* = 18)	French Bulldog (8/23); Chihuahua (5/28); mixed (3/145); Pug (1/7); Welsh Springer Spaniel (1/3)
Post-trauma (*n* = 12)	Dislocation (*n* = 5); collapse (*n* = 8); fracture (*n* = 2)	Labrador Retriever (3/62); mixed (2/145); Alaskan Malamute (1/4); Boxer (1/13); Chihuahua (1/28); Chow Chow (1/3); German Shepherd (1/21); Labradoodle (1/22); Welsh Springer Spaniel (1/3)
Aggressive bone lesion (n = 2)	Osteolysis; pathological fracture	Chihuahua (*n* = 1); Labradoodle (*n* = 1)

* The numbers between brackets represent the number of dogs of the breed with the condition/total number of dogs of the breed in the study population.

**Table 2:** Radiographic findings and breeds per category of sternal abnormality in cats.

Group	Findings	Breeds *
Intersternebral cartilage (*n* = 15)	New bone formation (*n* = 15); sclerosis (*n* = 5)	Domestic Shorthair (10/112); Bengal (1/3); British Shorthair (1/10); mixed (1/11); Norwegian Forest (1/4); Siamese (1/4)
Abnormal number (*n* = 27)	9 sternebrae (*n* = 7)	Domestic Shorthair (4/112); Bengal (1/3); mixed (1/11); Siamese (1/4)
	< 8 sternebrae (*n* = 20)	Domestic Shorthair (13/112); British Shorthair (2/10); Ragdoll (2/7); Oriental Shorthair (1/1); Persian (1/1); Sphynx (1/2)
Shape deformity (*n* = 8)	Pectus excavatum (*n* = 6)	Domestic Shorthair (6/112)
	Pectus carinatum (*n* = 2)	Domestic Shorthair (1/112); Norwegian Forest (1/4)
Post-trauma (*n* = 3)	Dislocation (*n* = 3)	Domestic Shorthair (3/112)

* The numbers between brackets represent the number of cats of the breed with the condition/total number of cats of the breed in the study population.

## Data Availability

Data is contained within the article.
